# Determinants of maternal health services utilization in Uganda

**DOI:** 10.1186/s12913-015-0943-8

**Published:** 2015-07-17

**Authors:** Gideon Rutaremwa, Stephen Ojiambo Wandera, Tapiwa Jhamba, Edith Akiror, Angela Kiconco

**Affiliations:** Makerere University, Centre for Population and Applied Statistics (CPAS), Kampala, Uganda; United Nations Economic Commission for Africa (UNECA), Addis Ababa, Ethiopia; United Nations Population Fund (UNFPA), Uganda Country Office, Kampala, Uganda; Uganda Bureau of Statistics (UBOS), Ministry of Finance, Planning and Economic Development (MoFPED), Kampala, Uganda

**Keywords:** Determinants, Maternal, Health services, Utilization, Uganda

## Abstract

**Background:**

Uganda’s poor maternal health indicators have resulted from weak maternal health services delivery, including access to quality family planning, skilled birth attendance, emergency obstetric care, and postnatal care for mothers and newborns. This paper investigated the predictors of maternal health services (MHS) utilization characterized as: desirable, moderate and undesirable.

**Methods:**

We used a sample of 1728 women of reproductive ages (15–49), who delivered a child a year prior to the 2011 UDHS survey. A multinomial logistic regression model was used to analyze the relative contribution of the various predictors of ideal maternal health services package utilization. Andersen’s Behavioral Model of Health Services Utilization guided the selection of covariates in the regression model.

**Results:**

Women with secondary and higher education were more likely to utilize the desirable maternal health care package (RRR = 4.5; 95 % CI = 1.5-14.0), compared to those who had none (reference = undesirable MHS package). Women who lived in regions outside Kampala, Uganda’s capital, were less likely to utilize the desirable package of maternal health services (Eastern – RRR = 0.2, CI = 0.1-0.5; Western – RRR = 0.3, CI = 0.1-0.8; Central – RRR = 0.3, CI = 0.1-0.8; Northern – RRR = 0.4, CI = 0.2-1.0). Women from the richest households were more likely to utilize the desirable maternal health services package (RRR = 1.9; 95 % CI = 1.0-3.7). Residence in rural areas, being Moslem and being married reduced a woman’s chances of utilizing moderate maternal health care services.

**Conclusions:**

Utilization of maternal health services varied greatly by demographic and socio-economic characteristics. Women with a secondary and higher education, and those of higher income levels, were more likely to utilize the ideal maternal health services package. Therefore, there is need to formulate policies and design maternal health services programs that target the socially marginalized women.

## Background

Maternal health is a major global development challenge in Africa, which accounts for about half of the world’s maternal deaths, with little or no progress towards reduction of maternal mortality [1, 2]. Factors associated with maternal mortality in sub-Saharan Africa (SSA) were related to prenatal care coverage and skilled attendance at delivery [1]. On the other hand, failure to use maternal health services was a key predictor of perinatal death [3].

Early and frequent antenatal care (ANC) attendance during pregnancy is important to identify and mitigate risk factors in pregnancy and to encourage women to have a skilled attendant at childbirth. Postnatal care improves the health of both the newborn and mother. A study conducted in the Ulanga and Kilombero rural Demographic Surveillance sites in south-eastern Tanzania in 2008 [4], showed that poor quality of care, limited awareness about the health benefits of antenatal care, late recognition of pregnancy, and social and economic factors influenced timing of antenatal care [4–6].

In addition, ANC providers and the quality of services offered, play a key role in promoting delivery in health facilities [7]. Evidence from health facilities in rural Uganda, Tanzania and Burkina Faso showed that Health workers in all three country sites were unable to perform all procedures stipulated in the ANC guideline [8] irrespective of the availability of supplies. In addition, results from a Nepalese study revealed that a distance of more than one hour to the maternity hospital, low amenity score status, low education, multi-parity, and antenatal care during pregnancy were associated with an increased risk of home delivery [9, 10].

Levels of post-natal care in many countries are very low [11, 12]. Utilization of post-natal care (PNC) services is related to the level of awareness of the services, women’s occupation, ethnicity, the number of pregnancies and children, and the husbands’ socioeconomic status, occupation and education [11]. In addition, women experiencing health problems were strongly motivated to seek postnatal care. Also, women who had never used ANC, were less likely to use PNC, while those who had a smooth delivery, were less likely to use PNC services compared to those who had prior complications during delivery.

While there is a strong rationale for improving utilization of health services to improve maternal health, there are limited studies that identify factors affecting uptake of maternal health services, especially among vulnerable groups in Uganda. Previous Ugandan studies have only addressed one of the components of maternal health care and not all at ago [6, 13–15].

This study investigated the predictors of utilization of maternal health services encompassing prenatal, delivery care and post-delivery. Andersen’s behavioral model of health care services utilization, which has been widely applied on survey data and various health services and populations [16–18], was used to guide selection of covariates that could affect maternal health services (MHS) utilization. Therefore, the purpose of this study was to explore the association between selected socio-economic and demographic factors and the likelihood of utilizing moderate and desirable packages of MHS. This paper contributes towards enhanced understanding of factors that influence uptake of maternal health services in Uganda, based on a combined indicator of skilled attendance at delivery, antenatal care and post-natal care.

## Methods

### Data source

The 2011 Uganda Demographic and Health Survey (UDHS) data were used for this study. Authorization to use the data was obtained from MEASURE DHS by providing a brief description of the study through their website. Approval for UDHS data utilization for this study was obtained from the data originator, ICF Macro International U.S.A before the data was extracted from their web platform. At the point of data collection by the data originators, an informed consent was sought from all the study participants after detailed description of all the issues related to the study were passed across to the respondents. Eligible respondents who were unwilling to participate in the study were excluded from the survey. Consenting participants demonstrated their consent prior the commencement of the interview.

### Explanatory variables

In this study, Andersen’s behavioral model of health service use was adapted to examine the relationship between individual-level socioeconomic and demographic factors and the utilization of a specific package of maternal health services. According to Andersen’s behavioral model, healthcare utilization is a function of three major elements: predisposing factors (socio-demographic factors), enabling factors (e.g. income and health insurance) and healthcare needs such as functional disability and chronic illnesses [13, 14, 16]. The predisposing factors in the model were: age, education level attainment, region of residence, type of place of residence, religion, marital status and number of children ever born. Wealth indicator was considered an enabling factor, while visiting the health facility in the year prior to the survey was included in the model as a proxy for the need factor.

### Outcome variable

The UDHS had a set of questions on maternal health services. These included: number of ANC visits made by a woman pertaining to the last birth, place and provider of ANC, timing of first ANC visit, components used to assess the quality of ANC, tetanus toxoid, place of delivery, provider of assistance at delivery, timing of first PNC check-up, and the provider of the PNC check-up. We used these questions to generate an indicator outcome variable called maternal health services package. The probability that a woman would take a particular package of maternal health services as the dependent variable was assessed for all women.

The dependent variable had three (3) categories namely: ideal or desirable MHS package (coded as 1), moderate MHS package (coded as 2) and undesirable MHS package (coded as 3) as summarized below:

1. In the ideal/desirable category, women attended at least 4 ANC visits; they were assisted by a skilled personnel; delivered in a health facility and took their first post-natal check-up within 2 days following delivery;
2. In the moderate category, women received less than 4 ANC visits; had supervised delivery; delivered from a health facility; and received post-natal care, or had at least 4 ANC visits but did not receive any of the other components – supervised delivery in a health facility as well as PNC; Women who had received no ANC visits but received other components of care were also included in this category.
3. In the undesirable category, women made no ANC visits, did not receive delivery and PNC dimensions of care and did not deliver from a health facility.

### Sampling

A total of 1,764 women age 15–49 who delivered twelve months prior to the UDHS survey, were selected. Out of the 1764, 1728 completed information on both the covariates and the outcome variable and were selected for the main analysis in this study. This sample was adequate for the analyses and comparisons and useful in the identification of socio-economic and regional foci that could guide maternal health policy interventions in Uganda. The choice of events belonging to the one year prior to the survey date was to avoid memory lapse of the respondents.

### Statistical analyses

All variables included at bivariate analysis, were analyzed in the multinomial logit model. A multinomial logistic regression model was used to estimate variations in the probability of uptake of maternal health services among women age 15 to 49. This model is a simple extension of the logistic regression that allows each category of an unordered response variable to be compared to an arbitrary reference category providing a number of logit regression models. When using multinomial logistic regression, the relative risk ratios were determined for all independent variables for each category of the dependent variable with the exception of the reference category, which is omitted from the analysis. The regression model was fitted to the data in order to explore the association between a set of independent variables explaining the likelihood of a woman receiving the “ideal/desirable” maternal health services package as opposed to being in all other categories. The form of the equation fitted to the data was as follows:

1 $$ \ln \frac{P\left({Y}_i=m\right)}{P\left({Y}_i=l\right)}=\alpha +{\displaystyle \sum_{k=1}^K{\beta}_{xm}{X}_{ik}={Z}_{mi}} $$

For a dependent variable (maternal health services utilization) that had 3 categories, is represented by *m* in the equation above, and this requires the calculations for *m-1* equations, one for each category relative to the reference category to describe the likelihood of a woman receiving the ideal maternal health services package and the independent variables. For the moderate category of the dependent variable, for example the following equation derived from the latter is then estimated:

2 $$ P\left({Y}_i=m\right)=\frac{ \exp \left({Z}_{mi}\right)}{1+{\displaystyle \sum_{h=2}^M \exp \left({Z}_{hi}\right)}} $$

In the multinomial logistic regression, the undesirable MHS package was chosen as the comparison category. The model parameter estimates and the attendant Relative Risk Ratios (RRR) for the multinomial logit model is that for a unit change in the predictor variable, the logit of outcome *m* relative to the reference group is expected to change by its respective parameter estimate given that the variables in the model are held constant. The RRRs can be obtained by exponentiation the multinomial logit coefficients (e^**coefficient**^), or by specifying the **rrr** option. The alpha threshold for significant results was set at *p* = 0.05 (95 %).

## Results

### Descriptive characteristics

The distribution of the study population is presented in Table 1. Fewer women (24 %) had secondary and higher education. Western followed by Eastern regions of Uganda exhibited the largest distribution of the sample (28 % and 27 %, respectively). More than three quarters (85 %) were either Catholic or Protestant). There was somewhat even distribution of the women by age group. Majority of the women were rural residents (84 %) and belonged to the poor wealth index (42 %). More than half of the women were married (86 %), and about half (50 %) had 1–3 children.

**Table 1:** Weighted percentage distribution of respondents by selected background characteristics and year of survey

Variable	Category	Number	Percent (%)
Education level attainment	None	217	12.3
	Primary	1,132	64.2
	Secondary +	414	23.5
Region of residence	Kampala	107	6.1
	Central	374	21.2
	Eastern	483	27.4
	Northern	313	17.7
	Western	487	27.6
Religion	Catholic	739	41.9
	Protestant	755	42.8
	Muslim	216	12.2
	All others	54	3.1
Age group	15-19	212	12.0
	20-34	1,287	73.0
	25-49	265	15.0
Rural/Urban residence	Urban	268	15.2
	Rural	1495	84.2
Wealth Index	Poor	762	42.3
	Middle	363	20.6
	Rich	639	36.2
Marital status	Never married	90	5.1
	Currently married	1,511	85.8
	Previously married	160	9.1
Children ever born	1-3	875	49.6
	4-6	538	30.5
	7+	351	19.9
Distance to health facility	Big issue	777	44.1
	Not a big issue	985	55.9
Visited health facility in past year	No - Did not visit	315	17.9
	Yes-Visited	1445	82.1
MHS package utilized	Undesirable	1,560	90.1
	Desirable	80	4.6
	Moderate	92	5.3

About 44 % of the study population indicated that distance was an issue in their utilization of health services. A smaller proportion (about 18 %) of the women did not visit a health facility in the 12 months period prior to the UDHS survey of 2011. Finally, most (90 %) of the women utilized the undesirable MHS package.

Table 2 shows the bivariate relationships between some selected explanatory variables and the outcome variable. All variables except religion, age group and parity, were significantly associated (*p* < 0.05) with the outcome variable.

**Table 2:** Percentage distribution of respondents by selected background variables, and utilization of maternal health services in Uganda in 2011

Variable/category	Type of services package utilized			Number	Significance *(p)*
	Desirable	Moderate	Un-desirable		
Education Level					
None	95.3	1.6	3.1	217	
Primary	93.1	2.5	4.4	1,132	0.00
Secondary +	84.1	9.2	6.8	414	
Region					
Kampala	74.9	11.4	13.7	107	
Central	92.1	5.2	2.7	374	
Eastern	90.5	2.3	7.1	483	0.00
Northern	91.9	3.7	4.4	313	
Western	94.6	3.1	2.4	487	
Religion					
Catholic	91.3	3.4	5.3	739	
Protestant	91.3	3.9	4.8	755	0.08
Muslim	92.4	5.8	1.9	216	
All others	86.5	4.4	9.1	54	
Age group					
15-19	89.0	5.4	5.6	212	
20-34	91.3	3.7	5.1	1,287	0.53
35-49	93.2	4.1	2.7	265	
Residence					
Urban	81.2	8.3	10.5	268	0.00
Rural	93.1	3.2	3.8	1495	
Wealth Index					
Poor	93.9	1.8	4.3	762	0.00
Middle	92.9	2.4	4.7	363	
Rich	87.3	7.3	5.4	639	
Marital Status					
Never married	81.9	3.0	15.1	81.9	
Currently married	91.7	4.3	4.0	91.7	0.00
Formerly married	92.8	1.1	6.2	92.8	
Parity					
1-3	89.4	5.1	5.5	89.4	
4-6	93.2	2.0	4.8	93.2	0.08
7+	92.9	4.1	3.0	92.9	
Distance to health facility					
Big problem	94.1	2.8	3.1	94.1	0.00
Not a problem	89.0	4.9	6.1	89.0	
Visited health facility in past year					
Did not visit	95.6	2.9	1.5	95.6	0.01
Visited facility	90.3	4.2	5.5	90.3	

### Multivariate results

Table 3 presents the results of the multinomial logistic regression procedures. Education significantly influenced the utilization of the desirable package of maternal health services, and not the moderate services package. Holding other variables constant, having secondary or higher education increased the relative risk of utilizing desirable category of maternal health services (RRR = 4.5; 95 % CI = 1.5-14.0).

**Table 3:** Multinomial regression model predicting the Relative Risk Ratios of a woman utilizing Maternal Health Services (2011)

Variable/Category	Desirable MHS		Moderate MHS	
	RRR	95 % CI	RRR	95 % CI
Education Level				
None (rc)	1.00	-	1.00	-
Primary	2.28	[0.8-6.7]	0.74	[0.4-1.6]
Secondary +	*4.54	[1.5-14.0]	0.95	[0.4-2.3]
Region				
Kampala (rc)	1.00	-	1.00	-
Central	*0.33	[0.1-0.8]	*0.32	[0.1-0.8]
Eastern	*0.21	[0.1-0.5]	0.77	[0.3-1.7]
Northern	*0.45	[0.2-1.0]	*0.30	[0.1-0.7]
Western	*0.31	[0.1-0.8]	*0.25	[0.1-0.6]
Type of Residence				
Urban (rc)	1.00	-	1.00	-
Rural	1.13	[0.6-2.3]	*0.39	[0.2-0.7]
Religion				
Catholic (rc)	1.00	-	1.00	-
Protestant	1.17	[0.7-2.0]	0.80	[0.5-1.3]
Muslim	1.14	[0.6-2.3]	*0.24	[0.1-0.6]
All others	2.14	[0.7-6.4]	2.83	[1.0-8.3]
Marital Status				
Never married (rc)	1.00	-	1.00	-
Currently married	1.04	[0.4-2.8]	*0.40	[0.2-0.8]
Previously married	0.44	[0.1-2.0]	0.73	[0.3-1.9]
Wealth Index				
Poor (rc)	1.00	-	1.00	-
Middle	0.98	[0.4-2.2]	1.35	[0.7-2.6]
Rich	*1.93	[1.0-3.7]	0.96	[0.5-1.7]
Age (continuous)	1.02	[1.0-1.1]	0.94	[0.9-1.0]
Children ever born (continuous)	0.98	[0.8-1.2]	1.11	[0.9-1.4]
Distance to health facility				
A big issue (rc)	1.00	-	1.00	-
Not a big issue	1.93	[0.9-4.2]	*2.67	[1.2-5.9]
Visited health facility in past year				
Did not visit (rc)	1.00	-	1.00	-
Visited facility	1.45	[0.5-4.4]	0.52	[0.2-1.1]
Model Constant	*0.01	[0.0-0.1]	0.84	[0.1-5.8]

Note: rc = reference category; n = 1728; Wald chi-square = 170.2; pseudo R^2^ = 11.5 %; Base model = undesirable category

*Means that variable is significant at *p* < 0.05

Region of residence was also significantly associated with utilization of the desirable and moderate packages of maternal health services. The relative risk of utilizing desirable MHS package reduced for all other Ugandan regions compared to Kampala, the capital. Eastern Uganda exhibited the lowest log-odds of utilizing the desired category of maternal health services (RRR = 0.2; 95 % CI = 0.1-0.5).

The log-odds of utilizing moderate MHS reduced significantly among women who resided in rural areas compared to those in urban areas (RRR = 0.4; 95 % CI = 0.2-0.7). Similarly, the relative risk of utilizing moderate package of maternal health services, compared to the undesirable package decreased by a factor of 0.24 (95 % CI = 0.1-0.6) among Muslim women, compared to Catholic women. Furthermore, in comparison to women who were never married, married women were less likely to utilize the moderate package of maternal health services compared to the undesirable package (RRR = 0.4; 95 % CI = 0.2-0.8).

The findings in Tables 3 further show that the relative risk of a woman utilizing the desirable package of maternal health services relative to the undesirable package, increased by a factor of 1.9 (95 % CI = 1.0-3.7) among women in the rich wealth category, compared to those in the poor category. Finally, the log-odds of utilizing the moderate maternal health services’ package relative to using the undesirable package, increased among women who thought that distance was not a major factor in utilizing health care services (RRR = 2.7; 95 % CI = 1.2-5.9).

## Discussion

This study found out that women with secondary and higher education were significantly more likely to utilize the desirable maternal health care package compared to those who had none. Women who lived in regions of Uganda other than Kampala were less likely to utilize the desirable package of maternal health services, while women from the affluent households were more likely to utilize the desirable maternal health services package. Similarly, for rural residence, being a Muslim and being married significantly reduced the chances of utilizing moderate maternal health services package. In addition women who thought that distance to the health facility was not a major factor were more likely to utilize the moderate package of maternal health services.

Uganda’s maternal mortality ratio has remained persistently high at 418 per 100,000 births in 2006 and 438 per 100,000 births by 2011. This is because a high proportion of women do not deliver under supervision in health facilities. In 2011, an estimated 42 % of women did not deliver from health facilities, reducing from 58 % in 2006 [19]. According to the UDHS 2011, 95 % of mothers received antenatal care from a skilled provider, but only 48 % of the women made four or more antenatal care visits, and this percentage remained almost the same since 2006. Only one third of women received postnatal care in the first two days after delivery, and for births in the two years preceding the survey, while only 2 % received a postnatal checkup within one hour, while 13 % received a postnatal checkup within six days.

The current study found that 90 % of women utilized undesirable package of health services. It appears that examining each of the components of the health care package obstructs the true picture of the situation concerning those women that utilize the full complement of the recommended maternal health services. The results have revealed that women who are likely to utilize the ideal package of maternal health services are the rich and those who have a secondary and higher education, which supports the current finding.

In a facility based study in Busia, a rural district in Eastern Uganda, the eight independent predictors that favored delivery in a health facility included: being of high socio-economic status, previous difficult delivery, parity less than four, preference of supine position for second stage of labor, preferring health workers to dispose the placenta, not having difficulty with transport, being autonomous in decision to attend antenatal care and depending on other people (e.g. spouse) in making a decision of where to deliver from [20]. We note, however, that the choice of childbirth site much as it depends on the pregnant woman, is often associated with health workers’ attitudes, locations of the sites, the antenatal clinic sites, the availability of supplies and drugs, and the availability of emergency obstetric care [21].

A study to investigate the factors associated with the utilization of modern and professional childbirth using the 2006 UDHS survey data showed that secondary or higher education was the strongest predictor of utilization of childbirth care [22]. Whereas partner’s education was important, maternal education in itself exerted a much stronger association with utilization of desired maternal health services package. Other factors associated with utilization of supervised professional delivery care included: community infrastructure, occupation, location, and regional differences, wealth status, religion, and age cohorts.

Cultural values and norms also influence choice of the place of delivery. For example, among the Sabiny of Eastern Uganda, cultural beliefs and practices are a key factor influencing choice of place of birth. Majority of the women opt for homebirths, and about quarter of them deliver at health facilities [23]. Ugandan women adhere to very traditional birthing practices and believe that pregnancy is a test of endurance and maternal death is merely a sad but normal event. A study conducted in Kiboga district of Uganda showed that pregnancy and childbirth were one of the major areas where women still command power and status, which they would strive to use to enhance their status within the household and community [24]. The latter factor is also addressed in a study conducted in South Western Uganda, which suggested that women feel that they have the most power and control during the birthing process, which is something they often lack in other aspects of their lives [25]. Therefore, in some Ugandan societies, women are considered to be strong and independent if they can handle the birthing process by themselves. This cultural view prevents women from seeking professional maternity care.

Secondary or higher education of the women decreased the likelihood of utilizing either poor or moderate MHS package. Secondary educational substantially increased women’s usage of ideal maternal health services indirectly through enhancing her socio-economic status [26]. These educational benefits are preventive in nature and materialize before and during pregnancy and after child delivery [16, 17, 25, 26]. For example, lack of education can prevent awareness of life-threatening obstetrical complications, which in turn reduces women’s recognition of the need to seek risk-appropriate health care [27].

Women’s limited decision-making power, as well as constrained economic resources, can inhibit their ability to seek health services and/or contribute to delays in accessing and receiving medical care even in places where services are readily available. Moreover, economic inequities may lead to substandard care offered to impoverished women who ultimately have the highest likelihood of dying during pregnancy, delivery or immediately post-partum.

The link between wealth of household and health seeking behavior can be explained by the fact that those who have more wealth are more likely to afford to purchase the required health and have better health outcomes [28]. The relationship between wealth and health is well documented in literature [22, 28] and often suggests that the higher the wealth status of an individual, the higher their likelihood of seeking ideal maternal health services, including maternal health services. It further highlights the household’s willingness to pay the expenses that are related to health care use [27].

Regional differences in utilization of maternal health services could perhaps be explained by the differences in access to maternal health services, the varying socio-cultural contexts and economic opportunities available to women in the respective regions. Residence in Eastern Uganda depicted higher usage of moderate package of maternal health services compared to Kampala region of Uganda. At the same time, all regions of Uganda were more likely to utilize the undesirable maternal health services compared to Kampala, the capital city. The spatial disadvantages of rural regions combined with social and economic seclusion of some population groups, may have contributed significantly to the relative over-utilization of the undesirable maternal health services by women in rural regions of Uganda [29].

Often rural–urban differences in utilization of maternal health services reflect the existing differences in access to health services and opportunities as earlier mentioned [29]. The reason for rural–urban disparity in utilization of health services is not a new phenomenon, and can be explained by variations in access to maternal health resources, where urban location often tend to be more privileged relative to rural areas [10]. Other studies have, however highlighted poor maternal health outcomes associated with urban poverty [30], especially in the poor slum settlements associated with major urban centers.

### Study limitations

The major limitation of this study relates to the secondary nature of the data that were used. Invariably many events captured through a retrospective inquiry are often susceptible to recall bias and memory lapse. For example information asked concerning number of times of ANC attendance might not be remembered exactly, which could impact on precision of the study findings. Furthermore, the data used may not be fully independent of selectivity issues arising from reports of women of reproductive ages. Only reports of women who were alive at the time of the survey were obtained, because in populations where maternal mortality ratios are very high, women of high mortality risk could have succumbed to the force of mortality [31].

Other variables including attitudes that a woman can handle a birth herself and other cultural factors were not analyzed and they might have had an impact on the findings of this study. The health services utilization models address a broad spectrum of factor which this study did not cover. Enhancing utilization of desirable maternal health services requires increased investment in secondary education at the community level, and strengthening of the health systems and improvement of quality of care [32]. This study did not cover any of the later variables and therefore was limited in that respect.

## Conclusions

We aimed at investigating factors that are associated with utilization of maternal health services in Uganda. First, we note that utilization of ideal maternal health services varies greatly by demographic and socioeconomic characteristics. The findings show that higher levels of education, being in Kampala, and richer wealth status, are associated with increased utilization of desirable maternal health services package.

Therefore, to promote ideal maternal health services utilization, there is need to formulate policies and design programs, which target women with low education, outside Kampala and of poor wealth status. Special attention should also be paid to women in the lower income categories, and those with no education. Given the complex and varied settings within which the maternal health programs are offered in Uganda, it is important to view any interventions within their demographic, socio-economic, political and geographic settings. The nature and scope of the population groups that are most affected by poor maternal health service delivery needs to be understood. Any future studies must therefore address aspects of health systems and the quality of care.

## Abbreviations

MHS: Maternal health services
DHS: Demographic and Health Survey
ANC: Antenatal care
PNC: Postnatal care
MDG: Millennium Development Goals
MCH: Maternal and Child Health
NGO: Non-Governmental Organization
EmOC: Emergency Obstetric Care
CI: Confidence interval
UBOS: Uganda Bureau of Statistics

## References

[1] Alvarez J, Gil R, Hernandez V, Gil A (2009). Factors associated with maternal mortality in Sub-Saharan Africa: an ecological study. BMC Public Health.

[2] Ogwang S, Najjemba R, Tumwesigye NM, Orach CG. Community involvement in obstetric emergency management in rural areas: A case of Rukungiri district, Western Uganda. BMC Pregnancy Childbirth. 2012;12. 10.1186/1471-2393-12-20 PMC3378461 22455614

[3] Abel M, Francoise K, Dramaix-Wilmet M, Donnen P (2012). Determinants of maternal health services utilization in urban settings of the Democratic Republic of Congo - A Case study of Lubumbashi City. BMC Pregnancy Childbirth.

[4] Gross K, Alba S, Glass T, Schellenberg J, Obrist B (2012). Timing of antenatal care for adolescent and adult pregnant women in south-eastern Tanzania. BMC Pregnancy Childbirth.

[5] Sarker M, Schmid G, Larsson E, Kirenga S, De Allegri M, Neuhann F, Mbunda T, Lekule I, Muller O (2010). Quality of antenatal care in rural southern Tanzania: a reality check. BMC Res Notes.

[6] Kabakyenga JK, Östergren P-O, Turyakira E, Pettersson KO. Influence of birth preparedness, decision-making on location of birth and assistance by skilled birth attendants among women in south-western Uganda. PLoS One. 2012;7. 10.1371/journal.pone.0035747 PMC3338788 22558214

[7] Adjiwanou V, LeGrand T (2013). Does antenatal care matter in the use of skilled birth attendance in rural Africa: A multi-country analysis. Soc Sci Med.

[8] Conrad P, Schmid G, Tientrebeogo J, Moses A, Kirenga S, Neuhann F, Müller O, Sarker M (2012). Compliance with focused antenatal care services: Do health workers in rural Burkina Faso, Uganda and Tanzania perform all ANC procedures?. Trop Med Int Heal.

[9] Wagle R, Sabroe S, Nielsen B (2004). Socioeconomic and physical distance to the maternity hospital as predictors for place of delivery: an observation study from Nepal. BMC Pregnancy Childbirth.

[10] Gabrysch S, Campbell O (2009). Still too far to walk: Literature review of the determinants of delivery service use. BMC Pregnancy Childbirth.

[11] Dhakal S, Chapman G, Simkhada P, van Teijlingen E, Stephens J, Raja A (2007). Utilisation of postnatal care among rural women in Nepal. BMC Pregnancy Childbirth.

[12] Matijasevich A, Santos I, Silveira M, Domingues M, Barros A, Marco P, Barros F (2009). Inequities in maternal postnatal visits among public and private patients: 2004 Pelotas cohort study. BMC Public Health.

[13] Andersen RA: Behavioral Model of Families’ Use of Health Services. Chicago, IL; 1968. [Research Series]

[14] Andersen R, Aday L (1978). Access to medical care in the US: Realized and potential. Med Care.

[15] Andersen R, Newman JF (1973). Societal and individual determinants of medical care utilization in the United States. Milbank Meml Fund Heal Soc.

[16] Andersen RM. Revisiting the behavioral model and access to medical care: does it matter? J Health Soc Behav. 1995;1–10. 7738325

[17] Andersen RM (2008). National health surveys and the behavioral model of health services use. Med Care.

[18] Irfan FB, Irfan BB, Spiegel DA (2012). Barriers to Accessing Surgical Care in Pakistan: Healthcare Barrier Model and Quantitative Systematic Review. J Surg Res.

[19] UBOS, ICF International Inc: Uganda Demographic and Health Survey 2011. Kampala, Uganda; 2012.

[20] Anyait A, Mukanga D, Oundo GB, Nuwaha F. Predictors for health facility delivery in Busia district of Uganda: a cross sectional study. BMC Pregnancy Childbirth. 2012;12. 10.1186/1471-2393-12-132 PMC3514288 23167791

[21] Kkonde A, Dolamo BL, Monareng LV (2011). Ugandan women’s childbirth preferences. Afr J Nurs Midwifery.

[22] Bbaale E, Guloba A (2011). Maternal education and childbirth care in Uganda. Australas Med J.

[23] Kwagala B (2013). Birthing choices among the Sabiny of Uganda.

[24] Kyomuhendo G (2003). Low Use of Rural Maternity Services in Uganda: Impact of Women’s Status, Traditional Beliefs and Limited Resources. Reprod Health Matters.

[25] Kabakyenga JK, Östergren P-O, Turyakira E, Pettersson KO. Knowledge of obstetric danger signs and birth preparedness practices among women in rural Uganda. Reprod Health. 2011;8. 10.1186/1742-4755-8-33 PMC3231972 22087791

[26] Ricketts TC, Goldsmith LJ (2005). Access in health services research: The battle of the frameworks. Nurs Outlook.

[27] Ahmed S, Creanga AA, Gillespie DG, Tsui AO (2010). Economic status, education and empowerment: implications for maternal health service utilization in developing countries. PLoS One.

[28] Preston SH, Taubman P, Martin LG, Preston SH (1994). Socioeconomic Differences in Adult Mortality and Health Status. Demography of AGING.

[29] Rutaremwa G. Under-five mortality differentials in urban East Africa: a study of three capital cities. J African Popul Stud. 2012;26.

[30] Izugbara C, Ngilangwa D (2010). Women, poverty and adverse maternal outcomes in Nairobi. Kenya BMC Womens Health.

[31] Graham W, Brass W, Snow RW (1989). Estimating maternal mortality: the sisterhood method. Stud Fam Plann.

[32] Gage AJ (2007). Barriers to the utilization of maternal health care in rural Mali. Soc Sci Med.

